# Glycosides of *Cistanche* improve cognitive dysfunction in sleep-deprived model rats by activating the SIRT1/PGC-1α/Nrf2 signaling pathway

**DOI:** 10.3389/fnmol.2026.1945359

**Published:** 2026-10-07

**Authors:** Qiang Li, Shijun Zhao, Jiaojiao Huo, Yingzhen Wang, Yihong Sun, Jianxin Jia

**Affiliations:** 1 School of Basic Medicine and Forensic Medicine, Baotou Medical College, Baotou, China; 2 Department of Human Anatomy, Baotou Medical College, Baotou, China; 3 Key Laboratory of Human Anatomy at Universities of Inner Mongolia Autonomous Region, Baotou Medical College, Baotou, China; 4 Department of Critical Care Medicine, Baotou Central Hospital, Baotou, China

**Keywords:** cognitive dysfunction, glycosides of *Cistanche*, oxidative stress, sleep deprivation, synaptic plasticity

## Abstract

**Objective:**

Sleep deprivation suppresses the SIRT1/PGC-1α/Nrf2 signaling pathway, leading to oxidative stress, synaptic damage, and cognitive dysfunction. Glycosides of *Cistanche* (GCs) possess well-documented antioxidant and neuroprotective properties and have been reported to improve cognition in sleep-deprived animals, but the molecular mechanism remains unclear. We hypothesized that GCs alleviate sleep deprivation-induced cognitive dysfunction by activating the SIRT1/PGC-1α/Nrf2 signaling pathway.

**Methods:**

Sixty male Sprague Dawley rats were randomly assigned to six groups (*n* = 10): Control, Platform, Model, GCs, Control+GCs, and Estazolam (Est) groups. A 21-day sleep deprivation model was established using the modified multiple platform water method. Assessments included cognitive function by Morris water maze (MWM) test; Wake, non-rapid eye movement (NREM), rapid eye movement (REM), and total sleep time (TST) by Electroencephalogram (EEG); the serum levels of MDA, GSH-Px and SOD by ELISA, hippocampal CA1 neuronal morphology structure by Nissl staining and transmission electron microscopy (TEM), and the SIRT1/PGC-1α/Nrf2 signaling pathway and synaptic related proteins were detected by Western blot. In an additional cohort (*n* = 40), the SIRT1-specific inhibitor EX527 was used to validate the causal role of the pathway.

**Results:**

Compared with the Control group, the Model group exhibited significantly prolonged escape latency, along with reduced the number of platform crossings and the percentage time in the target quadrant (*p* < 0.05). The proportion of Wake significantly increased, while NREM, REM, and TST all significantly decreased (all *p* < 0.01). The serum level of MDA elevated, while GSH-Px and SOD activities markedly reduced (all *p* < 0.01). Hippocampal CA1 neurons showed disorganized arrangement, decreased Nissl bodies, and mitochondrial damage. Moreover, the SIRT1/PGC-1α/Nrf2 signaling pathway and synaptic related proteins significantly downregulated (all *p* < 0.01). Nevertheless, GCs intervention ameliorated cognitive dysfunction, alleviated oxidative stress and neuronal damage, and upregulated the protein expression levels above mentioned (all *p* < 0.01). Notably, these protective effects of GCs were abrogated by the SIRT1 inhibitor EX527.

**Conclusion:**

GCs inhibited oxidative stress damage and improved synaptic plasticity, and thereby alleviated sleep deprivation induced cognitive dysfunction by activating the SIRT1/PGC-1α/Nrf2 signaling pathway.

## 1 Introduction

Sleep deprivation, the state of not obtaining adequate sleep to maintain normal alertness and performance, has become a serious global public health problem owing to shift work, prolonged screen use, and social jetlag. Statistical data shows that Asian adults sleep less than 6.5 h per night, far below the recommended 7–9 h standard for European populations and experts (Watson et al., 2015; Willoughby et al., 2023). This situation is even more severe among impoverished populations in developing or developed countries (Rao et al., 2021). Chronic sleep deprivation induces metabolic disorders, immune dysfunction, and emotional disturbances such as anxiety and depression. Importantly, accumulating evidence indicates that sleep deprivation causes oxidative damage and synaptic structural and functional abnormalities in the brain, particularly the hippocampus, ultimately leading to cognitive dysfunction and accelerating the occurrence and progression of neurodegenerative diseases (Cao et al., 2020; Wu et al., 2024; Zhao et al., 2023). Elucidating the mechanisms by which sleep deprivation impairs cognition is therefore essential for developing effective interventions.

Mitochondrial dysfunction and oxidative stress imbalance are key pathological mechanisms mediating hippocampal damage and cognitive dysfunction caused by sleep deprivation (Zhang et al., 2023; Zhu et al., 2022). The silent information regulator 1(SIRT1)/peroxisome proliferator-activated receptor γ coactivator-1α (PGC-1α)/ nuclear factor-erythroid 2-related factor 2 (Nrf2) signaling pathway is the core pathway that regulates mitochondrial homeostasis, oxidative stress, and neuroprotection (Zhao et al., 2021). SIRT1 can activate PGC-1α through deacetylation, thereby promoting Nrf2 nuclear translocation and upregulating the expression of downstream antioxidant proteins, effectively clearing free radicals, reducing oxidative damage, protecting neuronal structure and function, and maintaining synaptic plasticity (Chen et al., 2024). It is reported that sleep deprivation can significantly inhibit the activity of the SIRT1 exacerbate oxidative stress and synaptic dysfunction, leading to cognitive impairment (Zuo et al., 2020).

Previous studies have shown that GCs have antioxidant, antiapoptotic, and improving cognitive function effects, and can alleviate the cognitive function of sleep deprivation model rats by regulating the expression of synaptic related proteins (Gu et al., 2016; Kan et al., 2021; Wang et al., 2020). However, there is currently no clear report on whether GCs exert neuroprotective effects by activating the SIRT1/PGC-1α/Nrf2 signaling pathway. Although Zuo et al. (2020) showed that sleep deprivation suppresses this axis and that upregulation of Sirt1 by hydrogen sulfide prevents hippocampal damage, it remains unknown whether natural products such as GCs restore this pathway in sleep-deprived animals and whether this pathway is causally required for their protective effects. We hypothesized that GCs alleviate sleep deprivation-induced cognitive dysfunction by activating the SIRT1/PGC-1α/Nrf2 signaling pathway. Based on this, this study aim to construct a sleep deprivation rat model by improving the multiplatform water environment method, to clarify the molecular mechanism of GCs improving cognitive dysfunction induced by sleep deprivation, and to provide new experimental evidence and therapeutic targets for the clinical prevention and treatment of sleep related cognitive impairment.

## 2 Materials and methods

### 2.1 Animals

Male SD rats aged 6–8 weeks, with a body weight of 265 ± 15 g, purchased from SPF (Beijing) Biotechnology Co., Ltd., with an animal production license [SCXK (Beijing) 2024-0001], were housed in the Animal Experiment Center of Baotou Medical College at a temperature of (24 ± 1) °C, humidity of 50–55%, and 12 h of alternating light/dark, with free access to food and water. Only male rats were used to exclude potential confounding effects of estrous-cycle-related hormonal fluctuations on sleep architecture, cognitive performance, and oxidative stress parameters. Animals aged 6–8 weeks (young adults) were selected to model the young adult population, in which insufficient sleep is most prevalent, and to avoid age-related neurodegenerative changes thatwould confound the interpretation of results. The experimental protocol was approved by the Animal Ethics Review Committee of Baotou Medical College [Approval No. (2023) 51].

### 2.2 Main reagents

GCs was purchased from Kosmai Plant Polysaccharide Development Co., Ltd., (Baoji, China) batch no. KSM20220609011; Estazolam tablets were purchased from Shiyao Group Ouyi Pharmaceutical Co., Ltd. (Shijiazhuang, China), NMPA approval no. 044220343; SIRT1, PGC-1α and Nrf2 primary antibodies were purchased from Abmart Medical Technology (Shanghai) Co., Ltd. (product number: TU365233S, T56630S, T55136S); SYN, PSD-95, BDNF, β-actin antibody were purchased from Proteintech Group Inc. (No. 17785-1-AP, 20665-1-AP, 28205-1-AP, 20536-1-AP); SIRT1 inhibitor EX527 was obtained from MedChemExpress (New Jersey, USA), batch no. HY-15452. All reagents were stored and prepared according to the manufacturers’ instructions.

### 2.3 Experimental methods

#### 2.3.1 Animal grouping and administration

Sixty rats were randomly divided into 6 groups (10 rats per group): Control, Platform, Model, GCs, Control+GCs, and Est. The Control, Model, and Platform groups were given 0.9% saline by gavage; the GCs group and the Control+GCs group were administered 50 mg/kg GCs by gavage, the dose was derived from the previous study of the research group (Wang et al., 2024); The Est group was given 0.54 mg/kg Est by gavage. Daily gavage/administration at 7: 00 a.m. for 21 consecutive days; subsequent validation experiments, 40 rats were randomly divided into 4 groups (10 rats per group): Control, Model, GCs, and EX527+GCs groups. The EX527+GCs group was administered 50 mg/kg GCs by gavage and 5 mg/kg EX527 by intraperitoneal injection (Ma et al., 2020).

#### 2.3.2 Establishment of the SD rat model

The preparation of the sleep deprivation model adopts an improved multiplatform water environment method. The specifications of the water tank are 130 cm × 50 cm × 45 cm, with a platform diameter of 5 cm for the Model, GCs, and Est groups and 10 cm for the Platform group. The water temperature is (20 ± 1) °C. Rats were adapted to the apparatus for 1 week before modeling (60 min per day); During the modeling period, sleep deprivation was performed on the platform from 8:00 to 20: 00 daily for 21 consecutive days, with free access to food and water (Çinar et al., 2023).

#### 2.3.3 Morris water maze test

(1) Positioning Navigation: Record the evasion latency within 120 s for 5 consecutive days; (2) Space exploration: On the 6th day, the platform was withdrawn and the number of passes through the platform within 120 s was recorded as a percentage of the time spent in the target quadrant (Chen et al., 2022).

#### 2.3.4 Electrode implantation surgery and EEG data analysis

After 5 days of adaptive feeding, rats were subjected to electrode (D-2628) implantation surgery, and postoperative recovery was 7 days. Rats were fasted for 12 h before surgery and anesthetized with 2% pentobarbital sodium (2 mL·kg^−1^). After the corneal reflex and pain reflex disappeared, the skull was exposed, and the electrode implantation positions (AP: +2.0 mm; ML: 0.0 mm; anterior fontanelle) and (AP: −3.8 mm; ML: −2.0 mm; inflection point) were located using a brain stereotaxic device. Electroencephalogram (EEG) electrodes were implanted into the positioning points, while electromyogram (EMG) electrodes were implanted into the neck muscles for collection (Hong et al., 2005). After surgery, the rats were housed in a single cage and given penicillin sodium for 3 consecutive days to prevent infection. EEG and EMG signals were collected using Medusa Tethered Acquisition System (Nanjing Bio-Signal Technologies Co., Ltd.) data acquisition software, and analyzed and organized using Sirenia sleep pro software. The sleep phases of rats were divided into wake phase, NREM phase, and REM phase, and the distribution time of each phase was calculated (Guan et al., 2004).

#### 2.3.5 ELISA detection of oxidative stress indicators

After deep anesthesia with 2% pentobarbital sodium (2 mL/kg), the whole blood was taken from the abdominal aorta and the serum was separated for subsequent ELISA analysis. The MDA, GSH-Px, and SOD levels were detected according to the instructions of the kits (Wuhan Saiweier Biotechnology Co., Ltd. no. G4306, G4302, G4310).

#### 2.3.6 Nissl staining

Nissl staining paraffin embedded brain sections were stained with 0.5% toluidine blue solution at room temperature for 10 min, washed the slices with distilled water. Slices were dehydrated using gradient ethanol series and then treated with xylene for transparency. The stained sections were observed under an optical microscope (Leica) to obtain images (Lv et al., 2026).

#### 2.3.7 Transmission electron microscopy

Transmission electron microscopy randomly selected 6 hippocampal tissues, fixed them, washed with PBS, and treated with 1% osmium acid. Then, gradient ethanol and acetone dehydration were performed, and acetone and 812 embedding agents were used to embed the hippocampal tissues. The samples were placed in a 60 °C oven for 48 h, sliced stained with 2% uranium acetate solution, washed, and stained with 2.6% lead citrate solution. Collected images with transmission electron microscopy, and observed the ultrastructure of mitochondria.

#### 2.3.8 Western blot

The animals were anesthetized with 2% pentobarbital sodium and the whole brain was taken. Use RIPA buffer and grinder to homogenize the tissue within 1 min, followed by freezing at −20 °C for 30 min. Centrifuge the tissue lysate at 12,000 rpm for 15 min at 4 °C and collect the supernatant. Use BCA protein assay kit (Thermo Fisher Scientific, United States) to determine protein concentration. Mix the protein sample with SDS-PAGE loading buffer in a 4:1 volume ratio and denature in a 100 °C metal bath for 10 min. Electrophoresis, membrane transfer, sealing, overnight incubation at 4 °C for primary antibody (SIRT1, PGC-1α, Nrf2, 1:1,500; SYN, PSD-95, BDNF, and β-actin antibodies 1:5,000.), room temperature incubation for secondary antibody (1:5,000), chemiluminescence imaging, quantitative analysis of band gray values (Shen et al., 2026). Western blot experiments were performed using hippocampal protein samples from *n* = 3 rats per group (independent biological replicates). Each protein sample was subjected to three technical replicate blots. The band intensity of each biological replicate was calculated as the mean value of its three technical replicates.

### 2.4 Statistical methods

All data were analyzed using two-way analysis of variance (ANOVA), followed by least significant difference (LSD) post-hoc tests to assess pairwise differences. Statistical significance was defined as a *p* < 0.05. All statistical analyses were performed using SPSS 31.0.0 software (IBM, Armonk, NY, USA). Rats that died during the 21-day sleep deprivation procedure or whose data were unusable (e.g., displaced EEG electrodes or movement-contaminated signals, insufficient or hemolyzed serum samples) were excluded from the corresponding analyses. The final number of rats included in each analysis is indicated in the corresponding figure legends and table captions (Figure 1).

**Figure 1:**
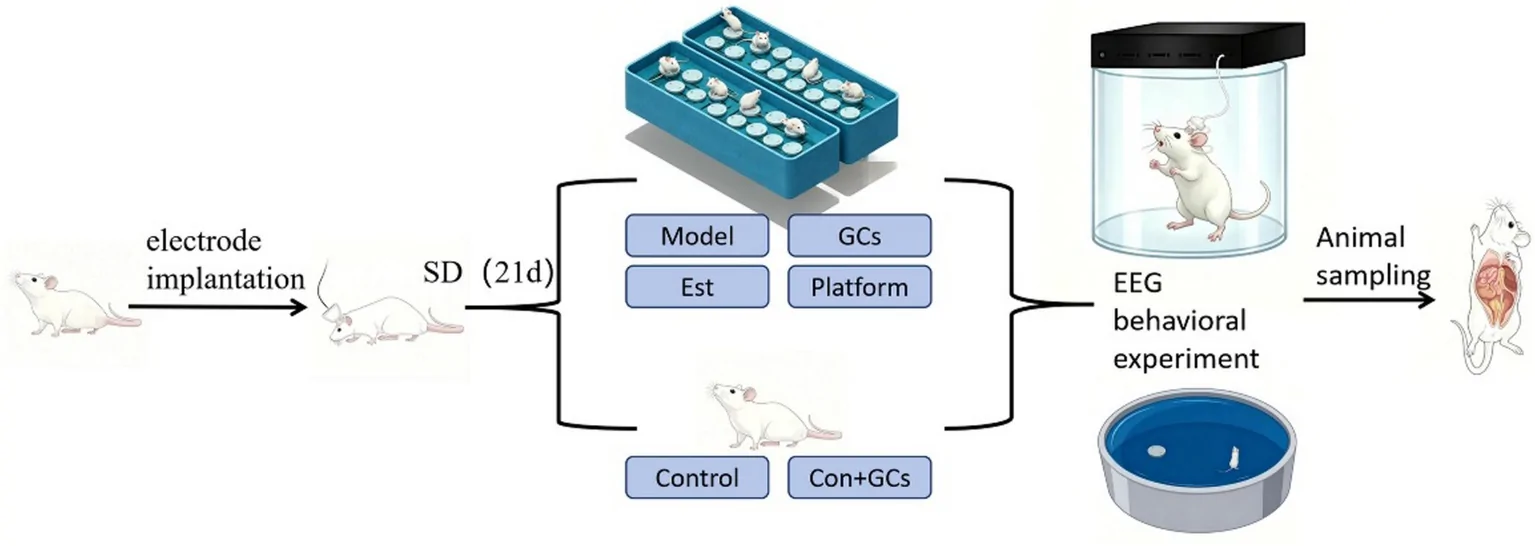
Diagrammatic representation of the experimental procedure.

## 3 Results

### 3.1 The effect of GCs on cognitive function in rats

Over the course of the 5-day positioning navigation training, both escape latency and path length for all groups exhibited a progressive decline accompanied by training days increased, suggesting that the swimming abilities of the animals were comparable at the outset. Beginning on the 2nd day, the two metrics for the control group rats decreased rapidly, whereas the model group rats demonstrated a significantly slower rate of decline compared to the control group. This finding suggests that the cognitive function of the model group was impaired, confirming the success of the modeling process.

The positioning navigation experiment showed that the escape latency and path length in each group all significantly reduced with the extension of training time. On the first day of training, the escape latency in the model group, GCs group, Est group and platform group was significantly shorter than that in the control group (*p* = 0.0267, *p* = 0.0012, *p* = 0.0278 and *p* = 0.0084, respectively). On the second day, the path lengths of the GCs group, Est group and platform group, as well as the escape latency of the GCs group and platform group, were significantly lower than those of the control group (all *p* < 0.05), while there was no difference between the model group and the control group. On the 3rd to 4th days, the path lengths of the GCs group and the platform group were significantly lower than that of the model group (all *p* < 0.05). On the third day, the escape latency in the GCs and Est groups was lower than that in both the control and model groups (*p* = 0.0238 and *p* = 0.0050, respectively) (Figures 2A,B).

**Figure 2:**
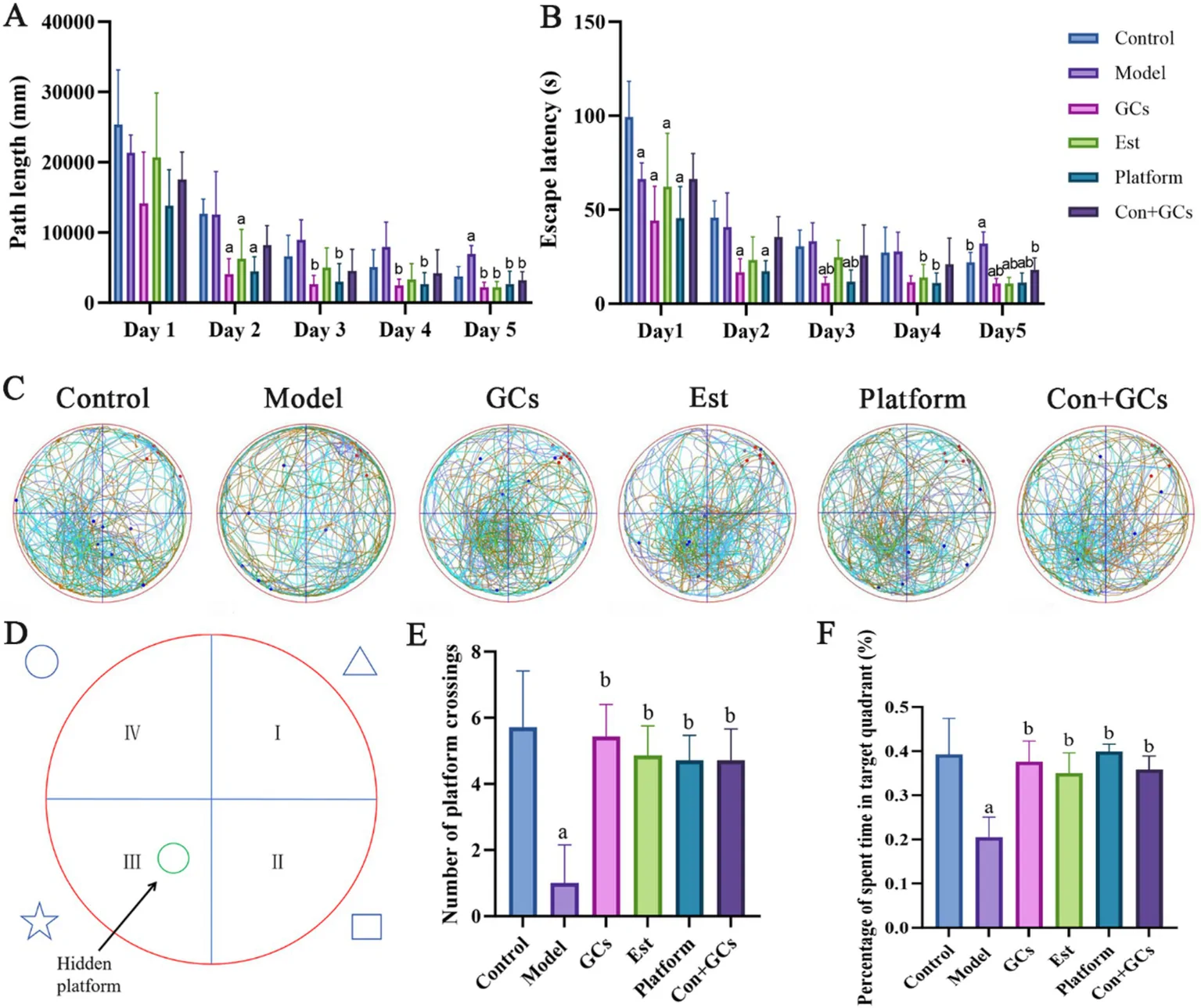
Effect of GCs on cognitive function by MWM test in sleep-deprived model rats (*n* = 8).**(A)** Path length, **(B)** Escape latency, **(C)** Swimming trajectory of space exploration experiments, **(D)** Schematic of the MWM, **(E)** The number of platform crossing, **(F)** Percentage of spent time in target quadrant. Compared with the control, ^a^*p* < 0.05; Compared with the model, ^b^*p* < 0.05.

The space exploration experiment demonstrated that, compared with the control group, the number of platform crossings and percentage of time spent in the target quadrant significantly decreased in the model group (all *p* < 0.0001, Figures 2C,D). In contrast, compared with the model group, the percentage of time spent in the target quadrant and the number of platform crossings all significantly increased in the GCs, Est, platform and Con+GCs groups (all *p* < 0.0001, Figures 2E,F). The results of the MWM test indicated that GCs significantly improved cognitive dysfunction induced by sleep deprivation.

### 3.2 The effect of GCs on sleep phase in rats

The total duration of each sleep stage within 2 h following sleep deprivation in all experimental group rats was quantified. Compared with the control group, the model group exhibited a significant increase in the proportion of Wake cumulative time, while the proportions of REM and NREM cumulative time significantly decreased (all *p* < 0.0001). In contrast, the GCs group demonstrated a significant reduction in the cumulative time ratio of Wake compared to the model group, accompanied by significant increases in the cumulative time ratios of REM and NREM (*p* = 0.0005, *p* = 0.0125, *p* = 0.005, *p* = 0.008, respectively), as illustrated in Figure 3.

**Figure 3:**
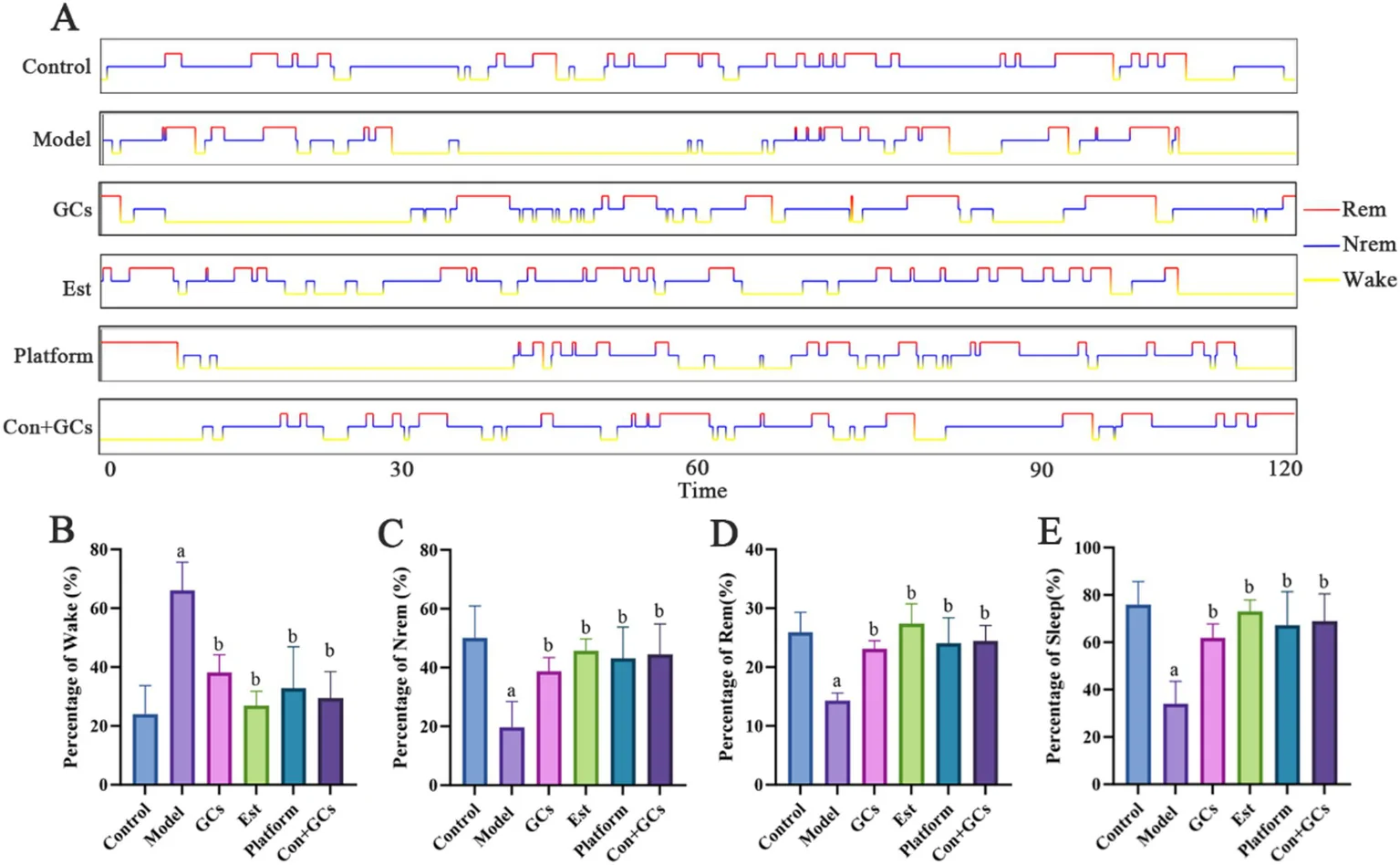
Effect of GCs on sleep phases in sleep-deprived model rats (*n* = 6). **(A)** Changes of sleep phases in different groups, **(B)** The proportion of Wake, **(C)** The proportion of NREM, **(D)** The proportion of REM, **(E)** The proportion of total sleep time. Compared with the control, ^a^*p* < 0.0001, Compared with the model, ^b^*p* < 0.05.

### 3.3 The effect of GCs on serum oxidative stress indicators in rats

ELISA results indicated that, compared with the control group, the activities of serum SOD and GSH-Px in the model group significantly decreased, while the content of MDA significantly increased (all *p* < 0.0001). Compared with the model group, the activities of serum SOD and GSH-Px in the GCs group significantly increased, and the content of MDA significantly decreased (all *p* < 0.0001). No significant differences were found between the GCs group and the Est group, as presented in Table 1.

**Table 1:** The serum levels of T-SOD, MDA and GSH-Px in each group (*n* = 9).

Group	T-SOD (U/ml)	MDA (μmol/L)	GSH-Px (U/ml)
Control	345.9 ± 10.76	3.470 ± 0.2475	1,958 ± 29.81
Model	208.9 ± 13.39^a^	6.217 ± 0.1953^a^	1,483 ± 56.74^a^
GCs	280.2 ± 23.70^b^	4.420 ± 0.2294^b^	1,780 ± 51.86^b^
Est	272.1 ± 24.36^b^	4.094 ± 0.5699^b^	1,771 ± 119.8^b^
Platform	289.7 ± 10.98^b^	4.201 ± 0.3034^b^	1,821 ± 87.72^b^
Con+GCs	310.5 ± 28.44^b^	4.017 ± 0.6115^b^	1,940 ± 29.62^b^

Compared with the control.

^a^*p* < 0.0001; Compared with the model.

^b^*p* < 0.001.

### 3.4 The effect of GCs on the morphology of hippocampal CA1 neurons in rats

The results showed that the control group, platform group, Con+GCs group and Est group all had a higher number of neurons and were rich in Nissl bodies. In contrast, the model group exhibited neuronal atrophy, with a significant reduction in the number of Nissl bodies and hippocampal neurons (*p* < 0.0001). Compared with the model group, GCs significantly improved the neuronal structure and the number of hippocampal neurons. No significant differences were found among the GCs, platform, Con+GCs, and Est groups. These findings suggested that GCs improved hippocampal neuronal damage resulting from sleep deprivation (Figure 4).

**Figure 4:**
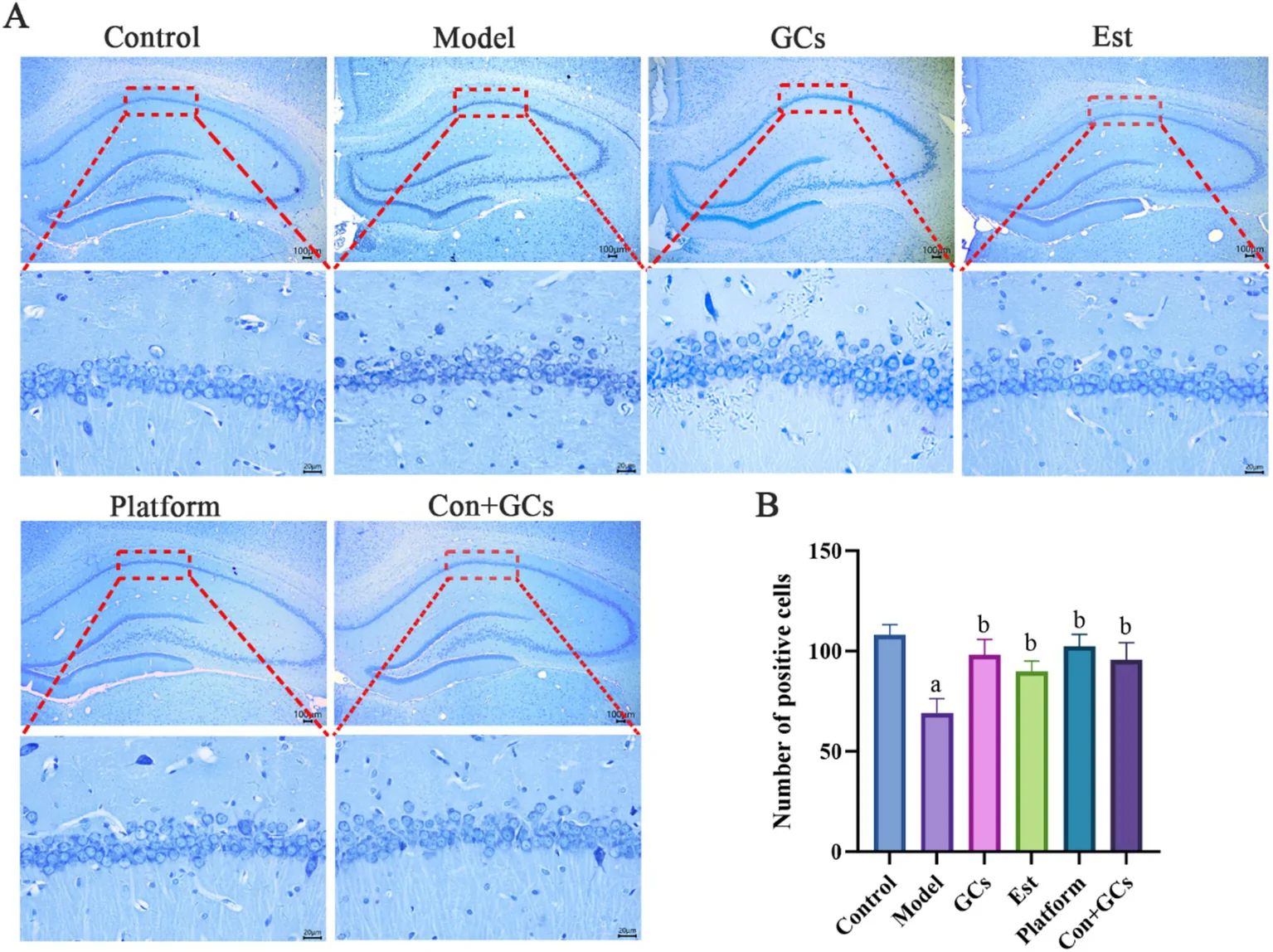
Effects of GCs on the neuronal morphology in the hippocampal CA1 region by Nissl staining (*n* = 3). **(A)** Microscopic field in hippocampal CA1 region (Above: 40×, below: 400×). **(B)** The number of hippocampal neurons in CA1 region. Compared with the control, ^a^*p* < 0.0001; Compared with the model, ^b^*p* < 0.0001.

### 3.5 The effect of GCs on the mitochondrial morphology of hippocampal CA1 neurons in rats

The results of transmission electron microscopy indicated that the number of mitochondria was relatively high in the control group, platform group, Con+GCs group, and Est group (*p =* 0.0003, *p =* 0.0037, *p =* 0.0007, *p =* 0.0007, respectively). In contrast, the model group exhibited unclear mitochondrial membrane boundaries, disrupted mitochondrial cristae, and the presence of vacuolation. The mitochondrial structure and damage conditions in the GCs group improved. No significant differences were found among in the GCs, platform, Con+GCs, and Est groups. These findings demonstrated that GCs could ameliorate mitochondrial damage induced by sleep deprivation. (Figure 5).

**Figure 5:**
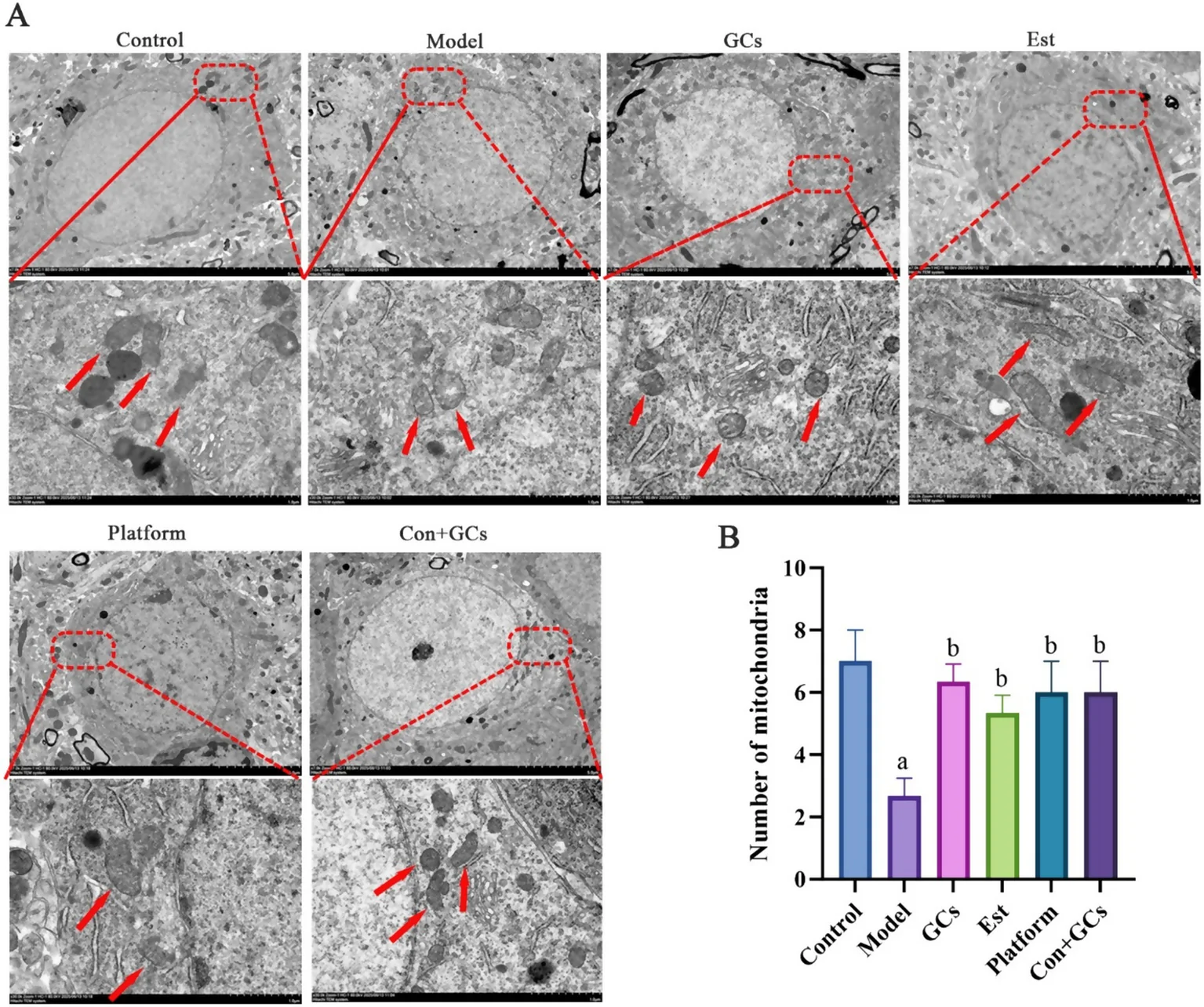
Ultrastructural observation of mitochondria of neurons in the hippocampal CA1 region in rats of each group by TEM (*n* = 3). **(A)** Mitochondrial ultrastructure field of view (Above: 7,000×, below: 30,000×). **(B)** The number of complete mitochondria in hippocampal CA1 region. Compared with the control, ^a^*p* < 0.0001; Compared with the model, ^b^*p* < 0.05.

### 3.6 The effects of GCs on the expression of synapse-related molecules and proteins in the SIRT1/PGC-1α/Nrf2 pathway

As shown in the Western blot results in Figures 6A–D, compared with the control group, the protein expression levels of SYN, PSD-95, and BDNF significantly decreased in the model group (all *p* < 0.0001), as well as the expression levels of SIRT1, PGC-1α, and Nrf2 significantly reduced (all *p* < 0.0001). In contrast, GCs administration up-regulated the expression levels of SYN, PSD-95, BDNF, SIRT1, PGC-1α, and Nrf2 compared to the model group (*p* = 0.0113, *p <* 0.0001, *p <* 0.0001, *p <* 0.0001, *p* = 0.0054, *p <* 0.0001, respectively, Figures 6E–H). No statistical differences were observed among the Est, platform, Con+GCs, and control groups.

**Figure 6:**
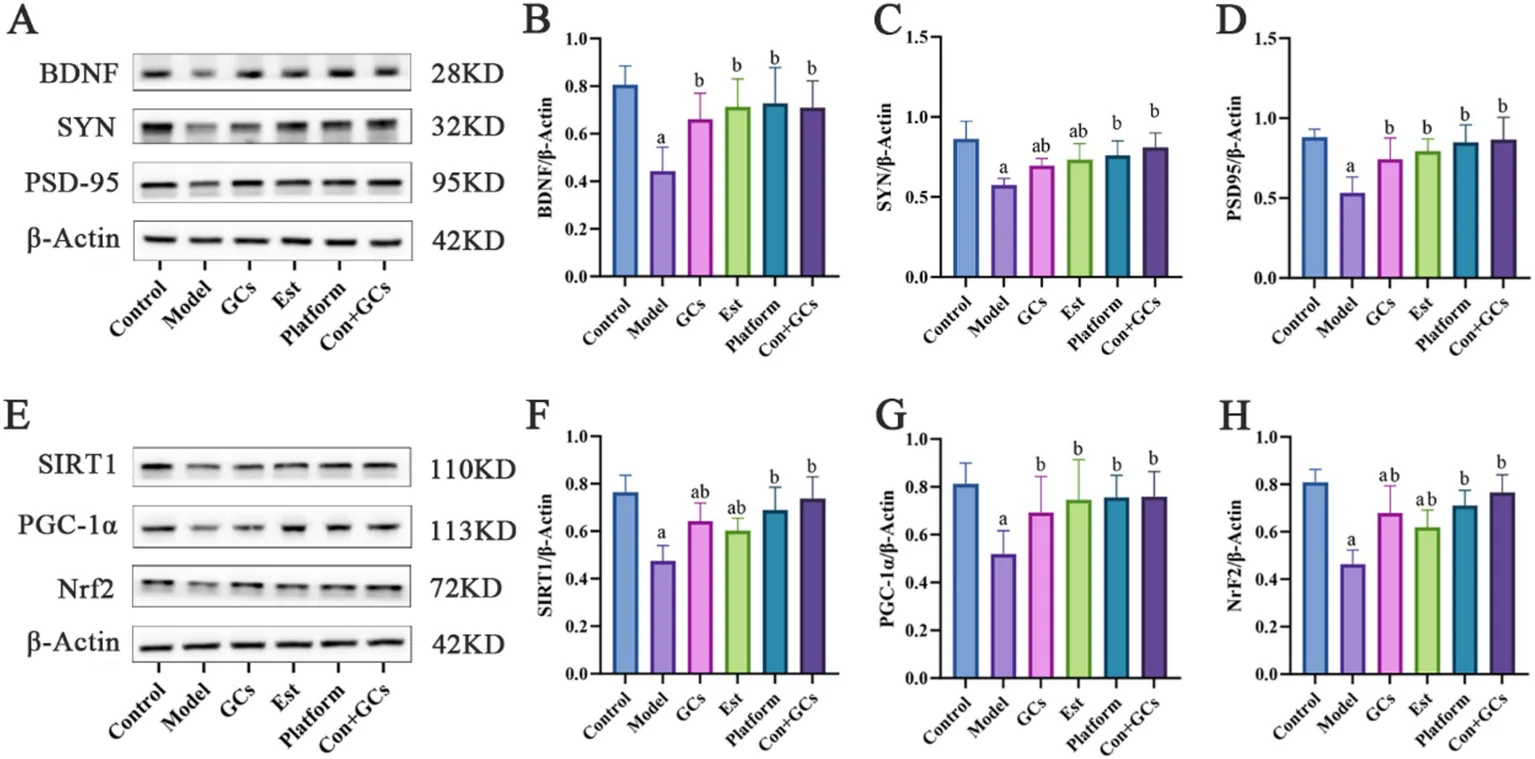
Effects of GCs on the protein expression levels of synapse-related molecules and the related factors of SIRT1/PGC-1α/Nrf2 signaling pathway (*n* = 3). Compared with the control, ^a^*p* < 0.0001; Compared with the model, ^b^*p* < 0.05.

### 3.7 The blocking effect of SIRT1 inhibitor EX527 on the synapse-related molecules and SIRT1/PGC-1α/Nrf2 signaling pathway

Compared with the GCs group, the intervention with EX527 (EX527 + GCs group) significantly prolonged path length and increased the escape latency, the number of platform crossings and percentage of time spent in the target quadrant significantly reduced (*p* < 0.001, *p* < 0.001, *p* = 0.0064, *p* = 0.0073, respectively), there were no significant differences in learning and memory ability between the EX527+GCs group and the model group (*p* = 0.47, *p* = 0.75, *p* = 0.9951, *p* = 0.9976, respectively, Figures 7A–F). Neurons in the hippocampal CA1 region exhibited disordered arrangement, reduced Nissl bodies, and structural damage such as mitochondrial cristae rupture and vacuolization (Figures 8, 9). The expression levels of SYN, PSD-95, BDNF, SIRT1, PGC-1α, and Nrf2 significantly downregulated (*p* = 0.0007, *p* = 0.0016, *p* < 0.0001, *p* = 0.0003, *p* = 0.0145, *p* < 0.0001, respectively, Figures 10A–H). The above results indicated that EX527 effectively blocked the improvement effect of GCs on cognitive dysfunction induced by SD.

**Figure 7:**
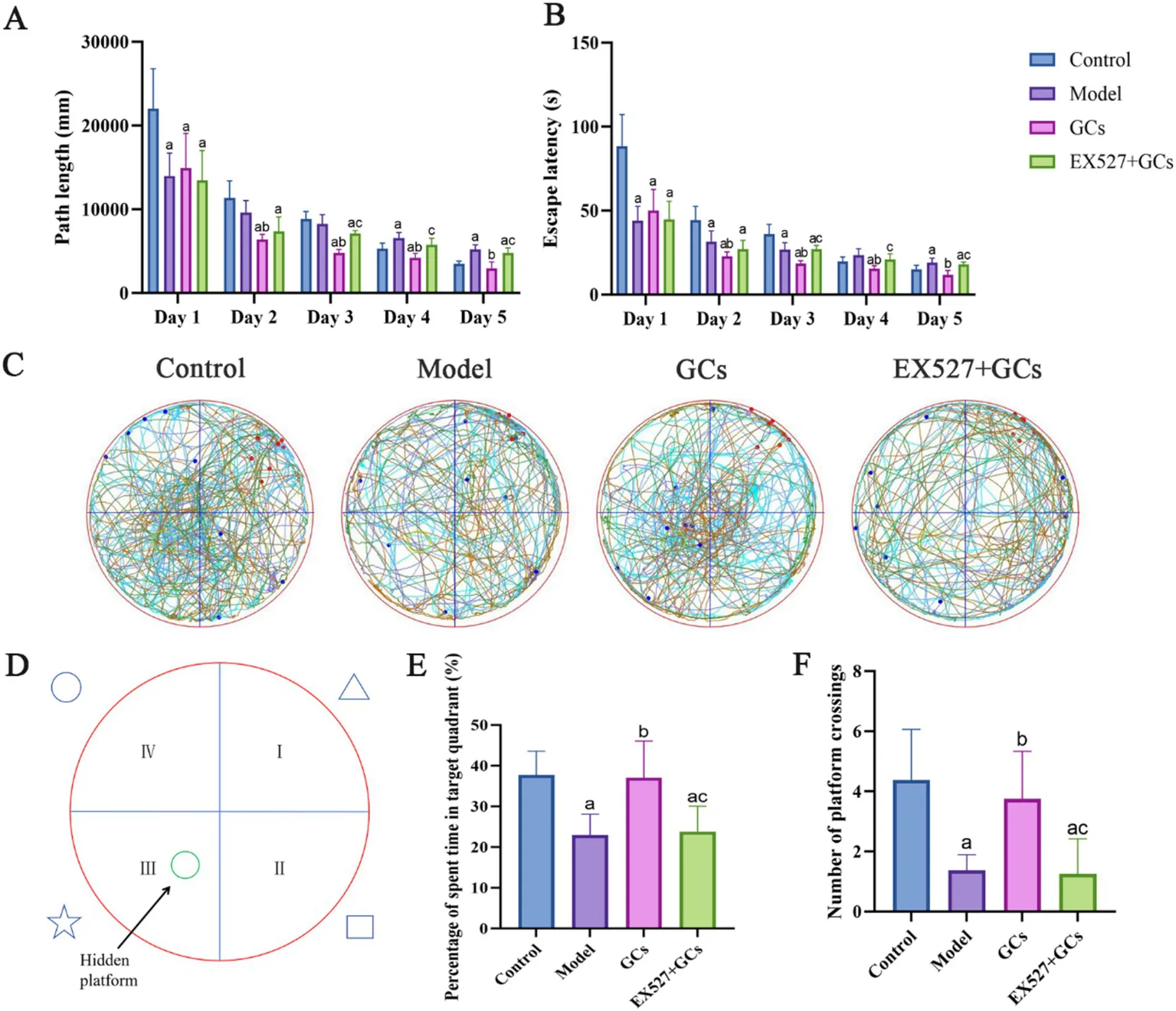
Effect of GCs on cognitive function by MWM test in sleep-deprived model rats (*n* = 8). **(A)** Path length, **(B)** Escape latency, **(C)** Space exploration experiments, **(D)** Schematic of the MWM, **(E)** The number of platform crossings, **(F)** Percentage of spent time in target quadrant. Compared with the control, ^a^*p* < 0.05; Compared with the model, ^b^*p* < 0.05; Compared with the GCs, ^c^*p* < 0.05.

**Figure 8:**
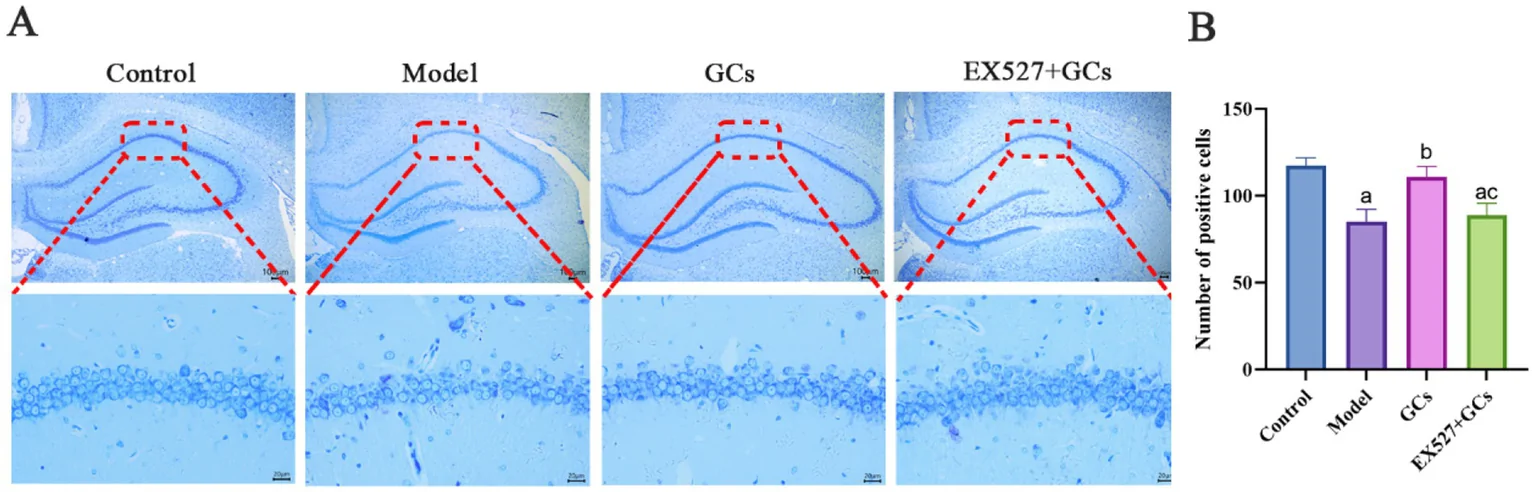
Observation of the morphology of neurons in the CA1 region of the hippocampus using Nissl staining (*n* = 3). **(A)** Microscopic field in hippocampal CA1 region (Above: 40×, below: 400×). **(B)** The number of hippocampal neurons in CA1 region. Compared with the control, ^a^*p* < 0.05; Compared with the model, ^b^*p* < 0.05; Compared with the GCs, ^c^*p* < 0.05.

**Figure 9:**
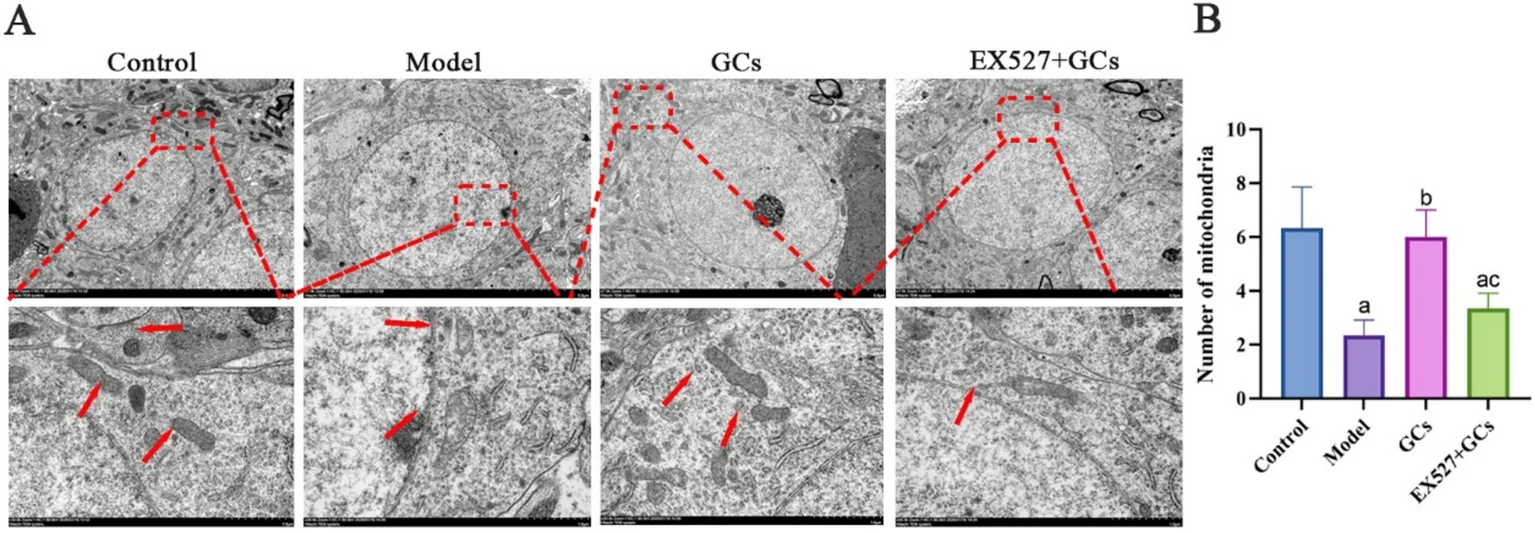
Ultrastructural observation of mitochondria of neurons in the hippocampal CA1 region in rats of each group by TEM (*n* = 3). **(A)** Mitochondrial ultrastructure field of view (Above: 7,000×, below: 30,000×). **(B)** The number of complete mitochondria in hippocampal CA1 region. Compared with the control, ^a^*p* < 0.05; Compared with the model, ^b^*p* < 0.05; Compared with the GCs, ^c^*p* < 0.05.

**Figure 10:**
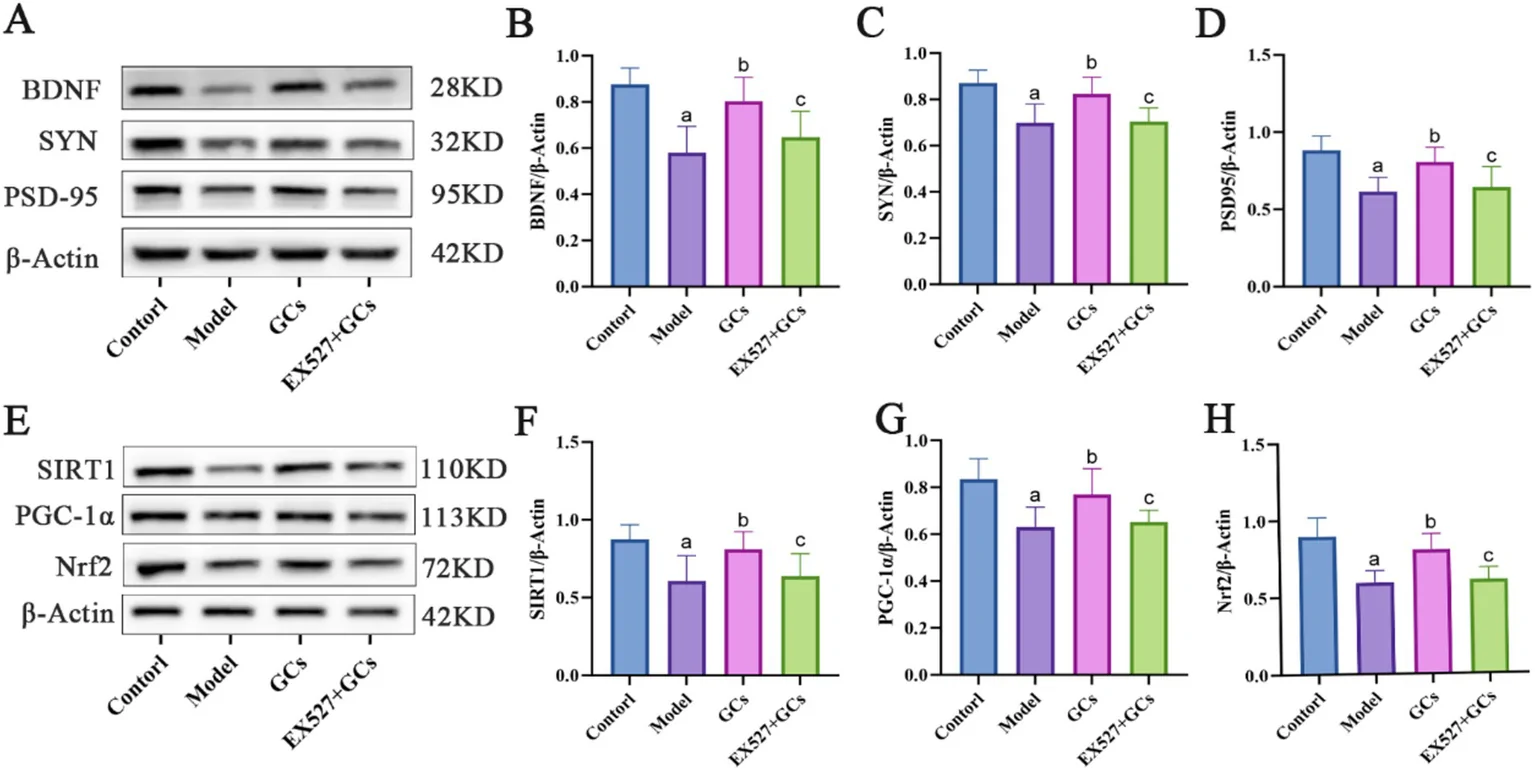
Effects of GCs on the protein expression levels of synapse‑related molecules and the related factors of SIRT1/PGC‑1α/Nrf2 signaling pathway (*n* = 3). (**A, E**) Representative western blot bands of target proteins. (**B–D**) Quantitative analysis of BDNF, SYN and PSD‑95 protein levels. (**F–H**) Quantitative analysis of SIRT1, PGC‑1α and Nrf2 protein levels. Compared with the control, ^a^*p* < 0.0001; Compared with the model, ^b^*p* < 0.005; Compared with the GCs, ^c^*p* < 0.05.

## 4 Discussion

It is suggested that sleep deprivation (SD) may cause central nervous system injury through multiple pathways (Kong and Zang, 2023). The present study further confirmed the pathological features of SD-induced neural injury. Specifically, SD rats exhibited a markedly decline in spatial learning and memory ability, accompanied by elevated serum levels of the oxidative stress marker (MDA) and reduced activities of the antioxidant enzyme (SOD) and (GSH-Px), these findings suggest that SD induces systemic oxidative stress imbalance (López-Armas et al., 2016). In our study, the pyramidal cells in hippocampal CA1 region displayed disordered arrangement, reduced Nissl bodies, ruptured mitochondrial cristae, and vacuolization, reflecting direct damage to neuronal architecture and mitochondrial integrity, the above results are consistent with previous report (Guo et al., 2021). Concurrently, the SIRT1/PGC-1α/Nrf2 signaling pathway was inhibited, and the expression levels of neurotrophic factor BDNF, as well as the synaptic proteins SYN and PSD-95 were down-regulated. Collectively, these results indicate that SD ultimately leads to cognitive dysfunction by suppressing antioxidant defense pathways and disrupting synaptic plasticity (Goyal et al., 2024; Liu et al., 2023). The above findings are consistent with previous studies, confirming that the SD model established in this study is reliable and suitable for further investigation of pharmacological intervention mechanisms.

GCs have been demonstrated to possess antioxidant, anti-apoptotic, and neuroprotective properties and to ameliorate cognitive impairment in SD animal models (Wang et al., 2024). However, the specific molecular targets underlying these effects remain unclear. The present study revealed that GCs intervention significantly reversed SD-induced cognitive deficits in rats, as evidenced by shortened escape latency and an increased the number of platform crossings in the MWM test. Furthermore, GCs treatment enhanced antioxidant enzyme activities, and alleviated pathological damage to hippocampal neurons and mitochondria. At the molecular level, GCs upregulated the expression of SIRT1/PGC-1α/Nrf2 pathway components and synaptic plasticity-related proteins. These results directly demonstrate that GCs mitigate SD-induced cognitive impairment through multiple mechanisms, including attenuation of oxidative stress, preservation of neuronal structure, and enhancement of synaptic plasticity, thereby broadening the recognized neuroprotection role of GCs. These findings are consistent with prior evidence that GCs modulate antioxidant pathways and improve neurological function.

The SIRT1/PGC-1α/Nrf2 signaling pathway is a central signaling axis that regulates oxidative stress, mitochondrial homeostasis, and neuroprotection (Gao et al., 2024; Zhu et al., 2023). SIRT1, an upstream NAD^+^-dependent deacetylase, activates PGC-1α and promotes Nrf2 nuclear translocation. This pathway not only exerts anti-inflammatory and antioxidant effects, but also reduces neuronal apoptosis, and preserves cellular function under pathological conditions (Huang et al., 2019; Villena, 2015). In the present study, SD significantly suppressed the protein expression levels of SIRT1, PGC-1α, and Nrf2, while treatment with GCs up-regulated the protein expression levels of SIRT1, PGC-1α, and Nrf2. These observations suggest that activation of the SIRT1/PGC-1α/Nrf2 pathway is a key mechanism underlying on the neuroprotective effects of GCs.

It is worth noting that Zuo et al. (2020) previously reported that sleep deprivation suppresses the SIRT1/PGC-1α/Nrf2 axis and that upregulation of Sirt1 by hydrogen sulfide protects the hippocampus. The present study extends this finding in several respects: we demonstrate that GCs restore the expression of the complete axis (SIRT1, PGC-1α, and Nrf2) together with the downstream synaptic proteins SYN, PSD-95, and BDNF; we provide causal evidence, using the SIRT1-specific inhibitor EX527, that this pathway is required for the protective effects; and we further show that GCs restore NREM and REM sleep architecture after sleep deprivation. These data identify GCs as a natural activator of this axis in sleep-deprived animals.

To further validate the specificity of this pathway, a SIRT1-specific inhibitor, EX527, was employed for confirmatory pharmacological blockade. The results demonstrated that EX527 reversed the GCs-induced improvements in cognitive function, oxidative stress injury, neuronal morphology, and pathway-related protein expression levels, indicating that the SIRT1/PGC-1α/Nrf2 pathway is required for, rather than merely accompanying, the protective effects of GCs. This pharmacological evidence confirms that the neuroprotective effects of GCs are dependent on SIRT1-mediated activation of the PGC-1α/Nrf2 pathway, thereby identifying the specific molecular target through which GCs ameliorate SD-induced cognitive impairment.

In summary, the present study provides convergent evidence from behavioral, histopathological, and molecular dimensions that GCs alleviate SD-induced oxidative stress injury, protect hippocampal neurons and mitochondria, enhance synaptic plasticity, and ameliorate cognitive dysfunction by activating the SIRT1/PGC-1α/Nrf2 signaling pathway. The reverse validation using the SIRT1 inhibitor EX527 further strengthens the scientific rigor of this mechanistic conclusion. These findings offer reliable experimental evidence supporting the potential clinical application of GCs for the prevention and treatment of cognitive decline associated with sleep disorders, and provide a reference for the future development of neuroprotective agents targeting the SIRT1 pathway (Figure 11).

**Figure 11:**
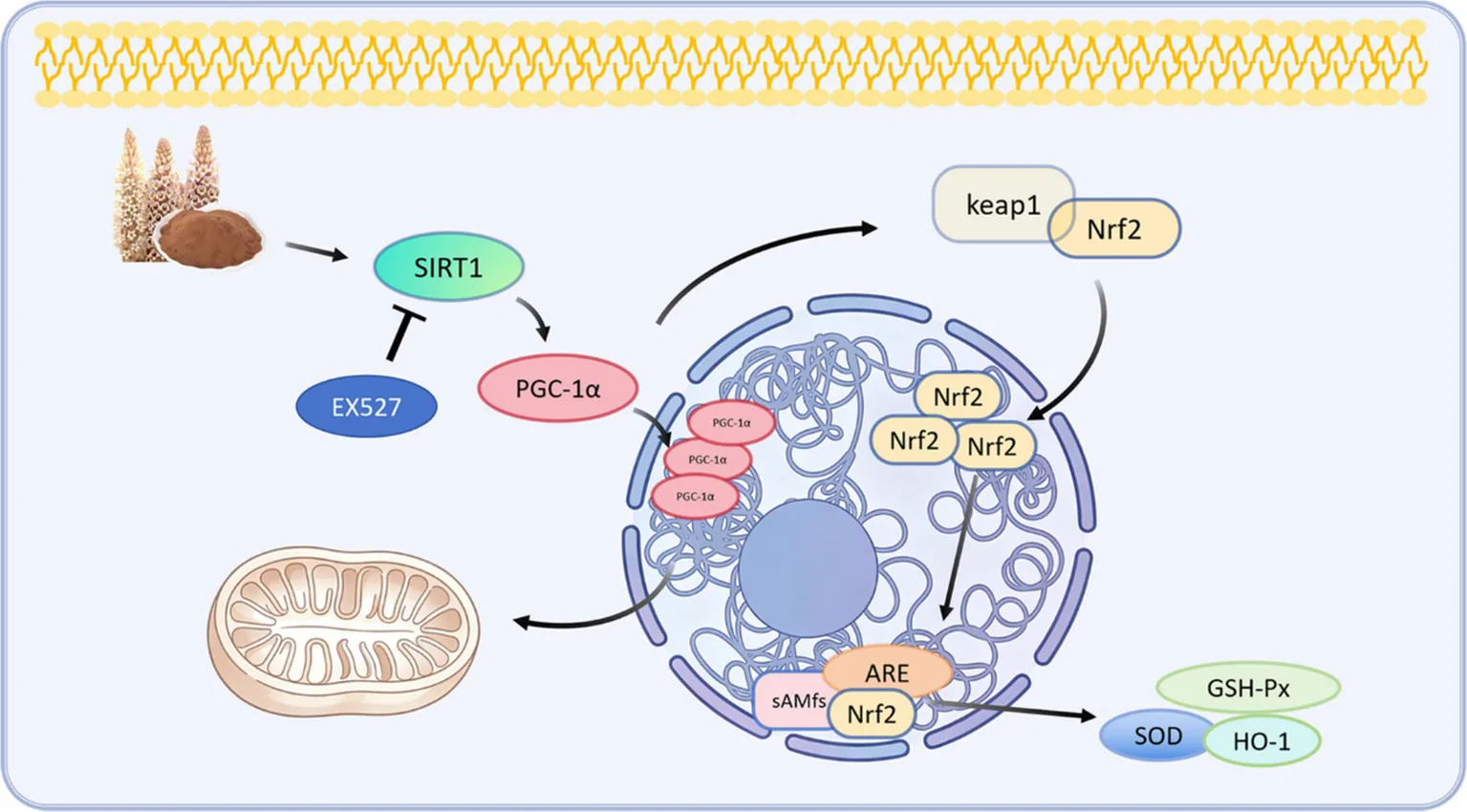
Specific mechanism by GCs improve cognitive dysfunction in sleep-deprived model rats via the SIRT1/PGC-1α/Nrf2 signaling pathway.

Several limitations should be acknowledged. First, only young adult male rats were used to minimize hormonal and age-related variability; whether these findings extend to female and aged animals remains to be determined. Second, a single dose of GCs was tested based on our previous study (Wang et al., 2024), and the dose–response relationship awaits systematic investigation. Third, GCs are a multi-component botanical extract, and the specific bioactive constituent(s) responsible for SIRT1 activation remain to be identified; more rigorous phytochemical standardization will facilitate clinical translation. Fourth, although pharmacological blockade with EX527 provides strong evidence that the SIRT1/PGC-1α/Nrf2 pathway is required for the protective effects of GCs, the possible contribution of additional pathways (e.g., direct Nrf2/Keap-1 activation or anti-inflammatory actions) cannot be excluded. Finally, sleep architecture was assessed only within a 2-h window immediately after sleep deprivation; longer-term polysomnographic monitoring in future studies would provide a more complete picture.

## 5 Conclusion

The present study demonstrates that GCs alleviate oxidative stress damage, preserve hippocampal neuronal integrity, enhance synaptic plasticity, and effectively ameliorate cognitive dysfunction in sleep-deprived model rats by activating the SIRT1/PGC-1α/Nrf2 signaling pathway. Pharmacological blockade with the SIRT1-specific inhibitor EX527 abolished the neuroprotective effects of GCs, further confirming that the SIRT1/PGC-1α/Nrf2 signaling pathway constitutes the core molecular mechanism by which GCs improve SD-induced cognitive dysfunction.

## Data Availability

The original contributions presented in the study are included in the article/Supplementary material, further inquiries can be directed to the corresponding authors.
